# InhA1-Mediated Cleavage of the Metalloprotease NprA Allows *Bacillus cereus* to Escape From Macrophages

**DOI:** 10.3389/fmicb.2018.01063

**Published:** 2018-05-23

**Authors:** Abbass Haydar, Seav-Ly Tran, Elisabeth Guillemet, Claire Darrigo, Stéphane Perchat, Didier Lereclus, Laurent Coquet, Thierry Jouenne, Nalini Ramarao

**Affiliations:** ^1^INRA, Micalis Institute, AgroParisTech, Université Paris-Saclay, Jouy-en-Josas, France; ^2^CNRS, UMR 6270, Normandy University, UNIROUEN, Plate-forme PISSARO, Mont-Saint-Aignan, France

**Keywords:** *B. cereus*, protein interaction, metalloproteases, macrophages, spores

## Abstract

*Bacillus cereus* is a Gram-positive spore-forming bacterium causing food poisoning and serious opportunistic infections. These infections are characterized by bacterial accumulation in the host despite the induction of inflammation. To circumvent inflammation, bacteria must resist the bactericidal activity of professional phagocytes, which constitute a first line of host defense against pathogens. Interactions between phagocytic cells and *B. cereus* are still poorly characterized and the mechanism of resistance to the host immune system is not known yet. We have previously shown that the spores are phagocytosed by macrophages but survive and escape from these cells. The metalloprotease InhA1 is a key effector involved in these processes. *inhA1*-deficient spores are retained intracellularly, in contrast to the wild type strain spores. NprA is also a *B. cereus* metalloprotease able to cleave tissue components such as fibronectin, laminin, and collagen. Here, we show that NprA, concomitantly secreted with InhA1 in the *B. cereus* secretome, is essential to promote bacterial escape from macrophages. We show that InhA1 cleaves NprA at specific sites. This cleavage allows liberation of the mature form of the NprA protein in the supernatant of the wild type strain. This mature form of NprA is actually the principal effector allowing bacterial escape from host macrophages.

## Introduction

Proteases are important virulence factors of pathogenic organisms during all steps of infection. They participate in the pathogen colonization of the host by degrading tissue extracellular matrix components like collagen and elastin. Their degradative activity helps bacterial proliferation by providing nutrients, interfering with the host immune system and damaging protective endothelial and epithelial barriers (Miyoshi and Shinoda, 2000).

*Bacillus cereus* is a Gram-positive spore-forming bacterium causing food poisoning and serious opportunistic infections (Stenfors Arnesen et al., 2008; Bottone, 2010; Decousser et al., 2013; Ramarao et al., 2014, 2015; Glasset et al., 2016, 2018; Lotte et al., 2017). The bacterium can survive in the host organism and generate infections despite the recruitment of phagocytic cells. The *B. cereus* genome comprises at least 50 genes coding for proteases with several putative functions during pathogenesis (Ivanova et al., 2003). Among them, two zinc proteases, InhA1 and NprA, were detected and quantified during several exoproteome studies (Clair et al., 2010; Madeira et al., 2015). In addition InhA1 is also associated with the spore exosporium (Charlton et al., 1999). InhA1 and NprA both contain the zinc-binding and catalytic active-site residues (HEXXH) common to metalloproteases. InhA1 is lethal when injected into the insect hemocoel, and is able to degrade antibacterial peptides such as cecropin and attacin (Dalhammar and Steiner, 1984). InhA1 is also involved in the capacity of the spores of *B. c*ereus to escape from host macrophages (Ramarao and Lereclus, 2005). InhA1 of *B. anthracis* (91% identity with *B. cereus* InhA1) is also secreted (Chitlaru et al., 2006) and digests various substrates, such as extracellular matrix proteins, and tissue components including fibronectin, laminin, and types I and IV collagens (Chung et al., 2006). InhA1 is involved in the modulation of blood hemostasis and thrombosis and in the increase of endothelial barrier permeability and hemorrhage (Mukherjee et al., 2011; Tonry et al., 2012). InhA1 is associated with altered levels of 92 *B. anthracis* proteins (Pomerantsev et al., 2011; Tonry et al., 2012; Pflughoeft et al., 2014). Thus, InhA1 plays a major role during virulence of *B. anthracis* by acting on bacterial and host proteins during infection.

NprA represents 60 to 80% of the *B. cereus* secretome in a minimum medium (Perchat et al., 2011). A *B. cereus* mutant deficient for *nprA* is as virulent as the wild type strain in an insect model of infection (Perchat et al., 2011) although *nprA* expression may be high in pathogenic *B. cereus* strains (Cadot et al., 2010). Thus, the exact role of NprA during pathogenesis is still unknown. Npr599, the homolog of NprA in *B. anthracis*, cleaves and activates the pro-urokinase plasminogen activator, a protein central in the fibrinolytic cascade, and its receptor. It also degrades the plasminogen activator inhibitors PAI-1 and PAI-2 (Chitlaru et al., 2006; Chung et al., 2006).

Macrophages are key players of the host immune response against bacterial infections. To successfully colonize their host, pathogens must defeat or avoid these cells. We have previously demonstrated that *Bacillus* spores are first internalized by macrophages but are able to escape, this capacity depending on the metalloprotease InhA1 (Ramarao and Lereclus, 2005). Here we show that NprA, which is concomitantly secreted with InhA1 in the *B. cereus* secretome, is essential to promote bacterial escape from macrophages. InhA1 regulates NprA at a post-transcriptional level by cutting NprA at specific sites. This cleaved form of NprA is a crucial effector promoting bacterial escape from host macrophages.

## Results

### InhA1 and NprA Are Concomitantly Secreted

**FIGURE 1:**
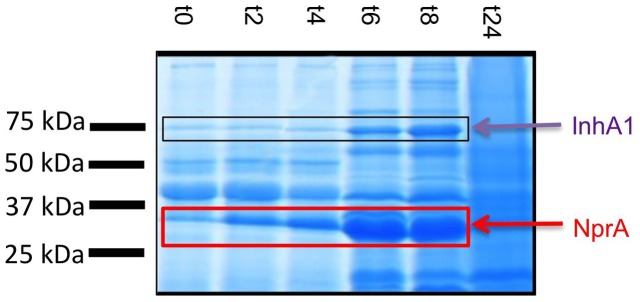
InhA1 and NprA throughout bacterial growth. Bc 407 strain was grown in NYB medium and culture supernatants were collected and filtered at the indicated time points. t0 indicates the point of entry of the culture into stationary growth phase. The proteins were precipitated and visualized on a SDS-Page gel. InhA1 and NprA were identified by Maldi-ToF.

The wild type Bc 407 strain was grown in NYB medium. At several time points during bacterial growth, the culture supernatant was harvested and filtered. Proteins present in the exoproteome were precipitated and visualized on a SDS-Page gel (**Figure 1**). Two proteins, detectable from the entry (t0) to the end (t8) of the stationary phase of growth, show an increase of intensity over time. The two proteins are not detectable at t24h. These two major proteins were identified by Maldi-ToF as InhA1 (apparent MW 75 kDa) and NprA (apparent MW 35 kDa).

### NprA Is Absent in the Secretome of the *inhA1* Mutant

**FIGURE 2:**
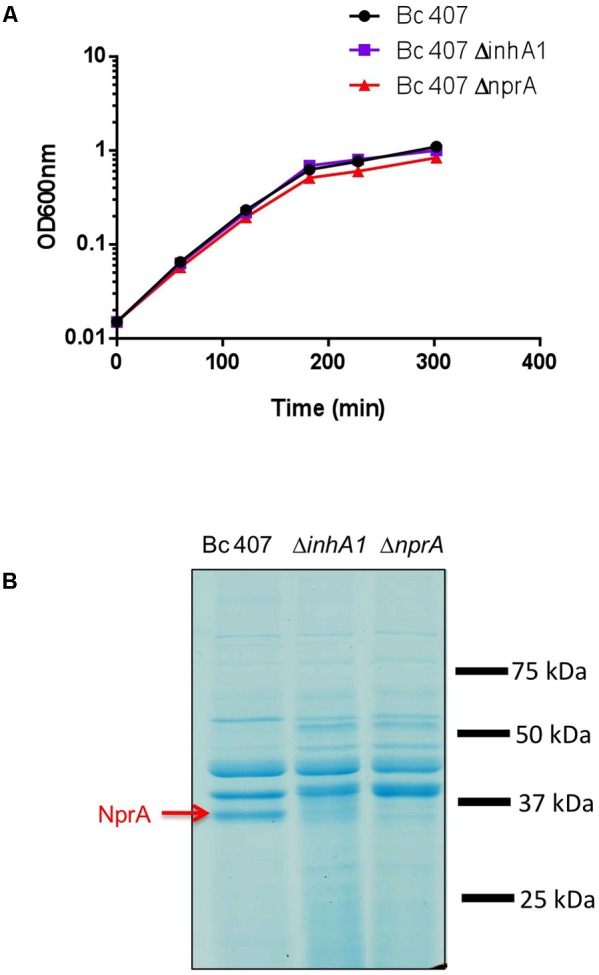
**(A)** Bc 407, Δ*inhA1*, and Δ*nprA* growth curves. Bc 407, Δ*inhA1*, and Δ*nprA* strains were grown in NYB medium and optical density was measured at 600 nm at the indicated times. The growth curves of the three strains were similar. The figure is representative of at last three different experiments. **(B)** NprA is absent in the supernatant of an *inhA1* deficient mutant. Bc 407, Δ*inhA1*, and Δ*nprA* strains were grown in NYB medium and culture supernatants were collected and filtered. The proteins were precipitated and visualized on a SDS-Page gel. NprA was identified by Maldi-ToF.

The growth curves of the wild type and the *inhA1* and *nprA* deficient mutants were similar (**Figure 2A**). The supernatant of the wild type and the *inhA1* and *nprA* deficient mutants were collected and proteins were separated by electrophoresis (**Figure 2B**). At the beginning of stationary phase (t2), NprA (MW 35 kDa) was present in the supernatant of the wild type strain but was absent in the supernatant of the *inhA1* deficient mutant. As expected, NprA was not found in the *nprA* deficient mutant.

**FIGURE 3:**
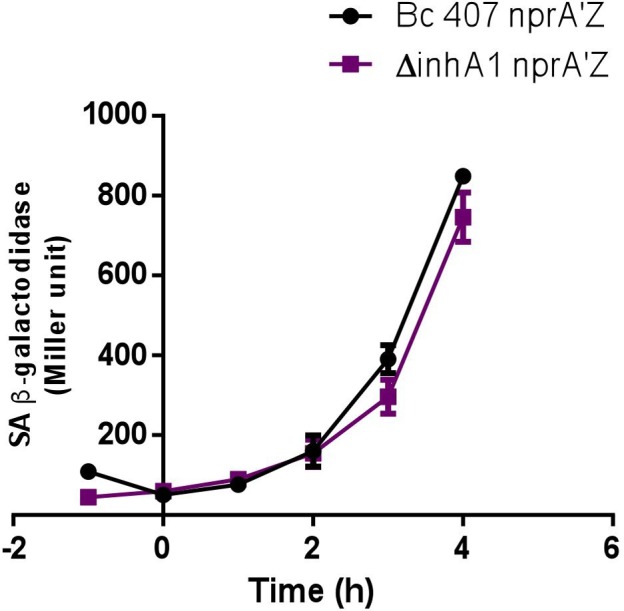
*nprA* gene transcription. The specific β-galactosidase activity (Miller unit) of strains Bc 407 and Bc 407 Δ*inhA1* harboring the transcriptional *nprA’Z* fusion were measured from bacteria grown in LB medium at 37°C from 1 h before the culture entry into stationary phase (t1) to 4 h after (t4). Results represent mean values of at least three independent experiments. No statistical difference could be determined (Mann–Whitney test).

We then assessed whether the absence of NprA in the *inhA1* mutant strain could be due to a regulation of *nprA* transcription by InhA1. However, no significant difference in *nprA* transcription could be observed between the wild type and the *inhA1* deficient strain (*P* > 0.15 at t4) (**Figure 3**).

**FIGURE 4:**
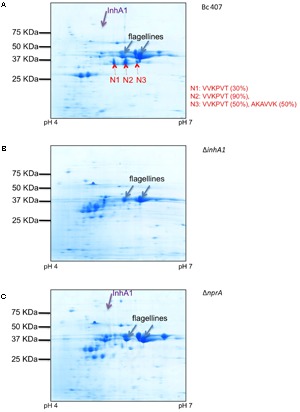
NprA in the supernatant of the wild type strain. Bc 407 **(A)**, Δ*inhA1* **(B)**, and Δ*nprA* **(C)** strains were grown in NYB medium and culture supernatants were collected and filtered. The proteins were precipitated and visualized on two-dimensional SDS-Page gels. NprA (N1, N2, N3, red arrows), InhA1 (purple arrows) and other proteins (i.e., flagellines, blue arrows) were identified by Maldi-ToF. The first six amino acids of the NprA fragments were identified by N-terminal sequencing (Edman degradation) and displayed in red. The percentage amount of the corresponding sequence found in each spot is indicated in brackets.

To visualize the NprA secreted by the different strains, the supernatant of the wild type strain and the two mutants deficient for *inhA1* and *nprA* respectively, were analyzed by 2D-gel electrophoresis and several proteins were analyzed by Maldi-ToF. Two major spots were identified as flagellines and were used to ensure the normalization of sample loading. Several proteins migrated around 28 kDa and were not visible in the 1D gels. These proteins were however, not analyzed. Several spots were analyzed by Maldi-ToF and identified as InhA1 and NprA. InhA1 was identified in the supernatant of the wild type (**Figure 4A**) and the *nprA* mutant (**Figure 4C**) strains but not in the *inhA1* mutant strain (**Figure 4B**). NprA was found in the wild type strain but not in the supernatant of the Δ*inhA1* and Δ*nprA* mutant strains. Several spots corresponding to NprA (indicated N1, N2, N3) were identified in the supernatant of the wild type strain. They all migrated around 35 kDa (**Figure 4A**). They were identified by Maldi-ToF as being NprA with log E values of -557, -470, and -638; protein coverage of 71, 72, and 72%, and 143, 190, and 186 spectra, respectively.

**FIGURE 5:**
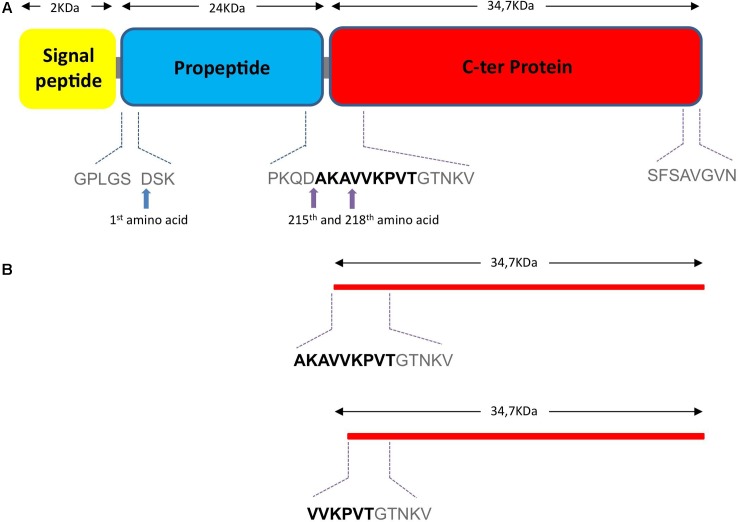
Theoretical 1D structure of NprA. **(A)** The theoretical 1D structure of the NprA protein was determined with the (http://www.ncbi.nlm.nih.gov/protein/143243?report=graph) site. Purple arrows indicate the InhA1 cleavage sites. **(B)** The two NprA forms produced following cutting by InhA are shown. First amino acid sequences are indicated.

Analysis of the three spots by N-terminal sequencing led to the identification of two main fragments starting with VVKPVT and AKAVVK, respectively. This corresponds to the amino acid position 215 and 218 in the full length NprA protein (without signal peptide), respectively (**Figure 5**).

Thus, InhA1 is necessary to allow the 35 kDa NprA fragments to be secreted in *B. cereus* supernatant.

### InhA1 Cleaves NprA at the Level of Its Propeptide

The NprA sequence is composed of a signal peptide, a sequence of 24 kDa, which may correspond to a propeptide, and a C-terminal domain of around 35 kDa (**Figure 5**). Thus, the presence of 35 kDa protein forms in the supernatant of the wild type strain and their absence in the supernatant of the *inhA1* deficient mutant suggests that InhA1 plays a role in the cleavage of NprA at the level of its propeptide.

**FIGURE 6:**
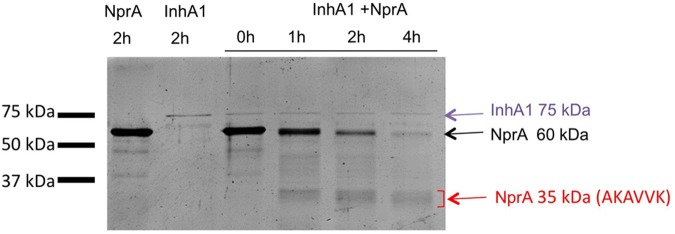
InhA1 cleaves NprA *in vitro*. Purified InhA1 and NprA (60 kDa) were incubated alone or with each other for 0 to 2 h before being loaded onto a SDS-PAGE gel. Proteins were identified by Maldi-ToF. The first six amino acids of the NprA fragments were identified by N-terminal sequencing (Edman degradation).

To confirm that InhA1 cleaves NprA, the two proteases were purified and their interaction was assessed *in vitro* (**Figure 6**).

InhA1 was purified as a recombinant His-tagged protein. NprA was purified first as a GST-tagged protein. The tag was then removed. Recombinant InhA1 and NprA migrated at 75 and 60 kDa, respectively following purification (lanes 1 and 2, **Figure 6**). Identity of InhA1 and NprA was confirmed by Maldi-ToF analysis (not shown). The purity of NprA was quite high although several bands appear at lower molecular weights, probably corresponding to degradation products of the protein. The cleavage of NprA by InhA1 was assessed by mixing 30 ng of purified InhA1 with 2 μg of NprA for 0 to 4 h. InhA1 signal was maintained over time, indicating that the protein was not cleaved nor degraded in the presence of NprA. By contrast, NprA was progressively degraded in the presence of InhA1. Several proteins appeared migrating around 35 kDa. Their analysis by Maldi-ToF and N-terminal sequencing confirmed the presence of C-terminal fragments of NprA. The main one corresponded to the protein produced in the supernatant of the wild type strain with the cleavage site (AKAVVK) (**Figure 4A**). The other fragment VVKPVT identified in the *B. cereus* supernatant was not detected *in vitro*, but the N-terminal analysis was not exhaustive, as several bands could not be sequenced. The quantity of NprA at 35 kDa did not increase, while the 60 kDa band disappeared over time following incubation with InhA1, suggesting that the whole NprA product is progressively degraded. Taken together, our data show that InhA1 cleaves NprA at various sites situated in close vicinity in the region between the propeptide and the theoretical active form of the NprA protein (**Figure 5**).

### NprA Allows Spore Escape Capacity From Host Macrophages

**FIGURE 7:**
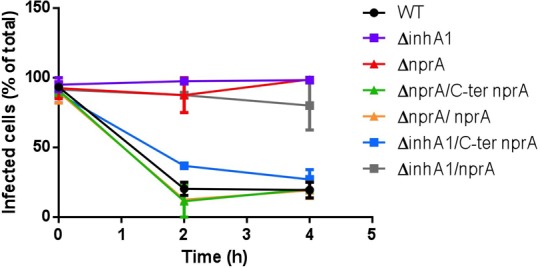
InhA1-mediated cleavage of NprA allows *Bacillus cereus* to escape from macrophages. J774 macrophages were infected for 30 min with spores of Bc 407 WT (black), Δ*inhA1* (purple), Δ*nprA* (red), Δ*nprA/C-ter nprA* (green), Δ*nprA*/*nprA* (yellow), Δ*inhA1*/C-ter *nprA* (blue), and Δ*inhA1*/*nprA* (gray) to allow phagocytosis. Non-phagocytosed spores were removed (t0) and fresh RMPI medium was added to infected cells for 0–4 h at 37°C under a 5% CO_2_ atmosphere. At the time points indicated, preparations were visualized under a light microscope and the percentage of cells containing at least one bacteria was calculated based on at least 300 cells per sample. Results are means of at least three experiments performed in duplicates for each strain and each time point.

We have previously shown that the *inhA1* mutant was impaired in its capacity to escape from macrophages after phagocytosis. Here, we have determined that InhA1 and NprA are produced concomitantly and that InhA1 cleaves NprA. We wished to determine whether NprA could play a role in macrophage escape. Macrophages were infected with *B. cereus* wild type spores and with mutants deficient for *inhA1* or *nprA* (**Figure 7**). After phagocytosis, all remaining extracellular spores were eliminated and cells were incubated for various times in fresh medium. At this time (t0) 80 to 100% of cells contained at least one spore for all the strains tested. Thereafter, the amount of infected cells decreased sharply when infected with the wild type bacteria and less than 20% of cells still contained intracellular bacteria at t4. In sharp contrast, mutants deficient for *inhA1* or *nprA* were severely impaired in their escape capacity with 100% of cells containing intracellular bacteria at t4 (*P* < 0.03 for both Δ*inhA1* and Δ*nprA* compared to wild type strain at t4). The *nprA* mutant, complemented with NprA was able to escape from macrophages with a phenotype similar to the wild type strain (*P* > 0.25). Thus, NprA, like InhA1, is required to allow *B. cereus* to escape from macrophages.

### InhA1-Mediated Cleavage of NprA Allows *B. cereus* to Escape From Macrophages

InhA1 cleaves NprA to a C-terminal form that could be the activated form of NprA. As InhA1 and NprA play a role during *B. cereus* escape from macrophages, we investigated whether the two proteases were acting together or independently. The escape capacity of the following strains was assessed: *inhA1* and *nprA* mutants complemented with either the full-length NprA protein or the C-terminal 35 kDa NprA.

As shown in **Figure 7**, when the *nprA* mutant was complemented with either the full length or the C-ter NprA, the spores were able to escape macrophages as efficiently as the wild type strain (*P* > 0.4). This strongly suggests that this cleaved-NprA promotes bacterial escape. To confirm this, the C-terminal 35 kDa NprA was inserted into the *inhA1* mutant. This strain was able to escape from macrophages as the wild type (*P* > 0.3). By contrast, *inhA1* mutant complemented with the entire NprA (60 kDa) was not able to escape from macrophages (*P* < 0.05). Thus, InhA1’s cleavage capacity on NprA is necessary to produce an active form of NprA able to allow the bacteria to exit the macrophages.

## Discussion

Zinc metalloproteases are widely distributed in nature and play important roles in many physiological processes. In the last 30 years, a collection of evidence has shown that a large number of these enzymes are multifunctional virulence factors playing pivotal roles in pathogenic interactions between bacteria and eukaryotic host organisms. Their hydrolytic activity exerted on a wide range of biologically important substrates results in facilitation of bacterial proliferation, through the provision of nutrients and invasion upon tissue destruction, and establishment of infection by silencing various host defense systems. This is the case of many well-studied bacterial zinc-dependent metalloproteases (Miyoshi and Shinoda, 2000). Among them, several thermolysins, have been cloned, purified, and characterized by crystallographic techniques. They are all synthesized as large propeptides, which are processed to mature proteolytically active forms. The maturation process is protein-specific but can be performed either intra or inter-molecularly (Miyoshi and Shinoda, 2000).

The theoretical structure of NprA presents a N-terminal domain, likely to be a propeptide, and a distinct C-terminal domain likely representing the active form of the protein following cleavage. NprA represents 60 to 80% of the *B. cereus* secretome in anaerobic minimal medium (Perchat et al., 2011). However, at the beginning of bacterial stationary growth phase, this protease is absent from the secretome of an *inhA1*-deficient mutant, showing that InhA1 plays a role in the modulation of NprA in the *B. cereus* supernatant. In this work, we show that InhA1 does not influence *nprA* gene transcription and that the modulation of the presence of the NprA protein in the secretome of the *inhA1* mutant is at the protein level. Consistently, we have also demonstrated that InhA1 cleaves NprA at specific locations of the protein allowing the secretion of C-terminal fragments of NprA in the supernatant of the wild type strain.

As InhA1 cleaves NprA *in vitro*, an uncleaved form of NprA was expected in the *inhA1*-deficient mutant. As the full length NprA could not be detected in the supernatant, we thus hypothesize that this 60 kDa form of NprA is either unstable in the supernatant of the *inhA1*-mutant or localized elsewhere in the bacteria. At the end of stationary growth phase, another C-terminal fragment of NprA of approximately 35 kDa is found in equal amount in the supernatant of the wild type and *inhA1*-mutant strains (not shown). Analysis of the NprA produced in the supernatants of both strains show that the protein sequence starts in both cases with VTGTNK. This implies that at the end of the stationary growth phase, proteases other than InhA1 cleave NprA in a similar cleavage area after the propeptide sequence. The differences in activity between the various forms of NprA remain to be studied.

Nowadays, very few studies have been undertaken on NprA importance for bacterial virulence. It has been shown that NprA is able to cleave tissue components such as fibronectin, laminin, and collagen, and thus displays characteristics related to pathogenic factors (Chung et al., 2006). Moreover, *nprA* gene regulation depends on the quorum-sensing system NprR/NprX (Perchat et al., 2011), which is implicated in necrotrophism (Dubois et al., 2012).

For successful infection, pathogens must defeat or avoid cells of the host immune system. Infections by virulent *Bacilli* are characterized by bacterial proliferation despite inflammation at the site of infection (Hernandez et al., 1998). This implies that the bacteria have developed means to resist to the inflammatory cells and thus to the host immune system. We have consistently shown that *B. cereus* is able to circumvent the host immune response (Tran and Ramarao, 2013; Darrigo et al., 2016; Guillemet et al., 2016). *B. cereus* spores survive, germinate, and multiply in contact with macrophages (Ramarao and Lereclus, 2005), eventually leading to the production of toxins responsible for macrophage death (Tran et al., 2011a,b; Ramarao and Sanchis, 2013). In this study, we show that NprA is a crucial element to counteract the host immune system by allowing the bacteria to escape from macrophages. Mutants deficient for *nprA* or *inhA1* are impaired in their escape capacity from macrophages. NprA ability to degrade host components may explain the role of this protein during macrophage escape. The cleavage of NprA by InhA1 is a pivotal element to promote NprA activity. Indeed, the active C-terminal domain of NprA is sufficient to promote bacterial escape from host macrophages after phagocytosis. Moreover, inability of the *inhA1* mutant to cleave a full length NprA prevents for bacteria ability to escape macrophages.

At present, we cannot exclude that any other protease could potentially be responsible for or contribute to bacterial macrophage escape. While our results strongly suggest a role of NprA in promoting bacterial escape, future experiments may further elucidate the mechanisms involved.

The cleavage capacity of InhA1 on *B. anthracis* proteins (including Npr599) has been recently observed (Pflughoeft et al., 2014). However, the cleavage sites were not determined and the physiological relevance of this cleavage during *B. anthracis* virulence was not studied. *B. anthracis* enters the host as spores. The first obligate step of infection is the capacity of the spores to cross the respiratory, digestive or cutaneous epithelial barriers. Indeed, the spores cannot actively cross the epithelial barrier and thus need to be captured by host cells to successfully invade the host. *B. anthracis* spores escape from the phagolysosome, germinate, and multiply within the macrophage cytoplasm, independently of the two virulence plasmids pX01 and pX02 (Dixon et al., 2000). There is to date no information on the role of InhA1 and/or NprA on the *B. anthracis* spore escape from macrophages.

Understanding the role that the primary phagocytic cells play in propagating the infection may be critical to decipher the mechanism by which the bacterium causes disease. In particular, a better knowledge on the role of proteases during pathogenesis will help to develop strategies to counteract these infections. These proteases could then be used as targets to develop new therapeutic approaches, targeting specific key points in the infection process.

## Materials and Methods

### Bacterial Strains and Growth Conditions

The Bc 407 Cry- (Bc 407) was used as a model for *B. cereus*. This strain cured of its plasmid is acrystalliferous and shows high phylogenic similarity with the *B. cereus* reference strain ATCC 14579 (Lapidus et al., 2008).

The mutant strains Bc 407Δ*inhA1* and Bc 407Δ*nprA* have been described previously (Grandvalet et al., 2001; Perchat et al., 2011).

All strains were grown in LB or NYB medium, which was prepared according to the manufacturer’s specifications at 37°C and bacterial growth was monitored by measuring the optical density at 600 nm. To prepare spores, strains were grown at 30°C in the sporulation-specific medium HCT + 0.3% glucose for at least 3 days until sporulation occurred. Spores were prepared as previously described (Ramarao and Lereclus, 2005).

### C-Terminal NprA Construction

A nucleotide construction was designed based on the Bc 407 genome (NCBI database, Reference No. NC_018877.1) and synthesized by the Eurofin Company. This construction assembled two regions bordering and including the *nprA* gene: the first one, corresponding to the promoter region of *nprA* and the signal peptide, ranges from the 606.964^th^ to the 607.207^th^ nucleotide and the second one, corresponding to the C-terminal part of NprA, ranges from the 607.823^th^ to the 608.797^th^ nucleotide. A *Bam*HI restriction site was added at the 5′ extremity of the construction, and a strop codon (TAA) followed by a *Sma*I restriction site was added at the 3′ end of the construction. This construction (C-ter NprA) was inserted into the pHT304 vector between the *Bam*HI and the *Sma*I restriction sites.

### Complementation of Bc 407Δ*nprA* and Bc 407Δ*inhA1* Strains

The pHT304 vector containing the complete *nprA* gene with its promoter region, or the C-ter NprA construction (starting AKAVVK) was used to transform Bc 407Δ*nprA* and Bc 407*ΔinhA1* strains by electroporation (Lereclus et al., 1989). Transformants were selected for resistance to erythromycin. The resulting new strains were designated Δ*nprA/nprA*, Δ*inhA1*/*nprA*, Δ*nprA/*C-ter*-nprA* and *ΔinhA1*/C-ter*-nprA*, respectively.

### Construction of Bc 407 *nprA*’Z and Bc 407Δ*inhA1 nprA*’Z Strains

The *nprA* gene was disrupted in the Bc 407 and the Δ*inhA1* strains by inserting a promoterless *lacZ* gene into the coding sequence as previously described (Perchat et al., 2011). In the resulting recombinant strains, the *lacZ* gene was transcribed from the *nprA* promoter. The strains were designated Bc 407 *nprA*’Z and Bc 407Δ*inhA1 nprA*’Z, respectively.

### Beta Galactosidase Assays

The β-galactosidase assays were performed as previously described (Guillemet et al., 2013; Tran et al., 2013). Briefly, bacteria were harvested from growing cultures at the indicated time points and disrupted with glass beads (Sigma). Subsequently, 2-nitrophenyl-β-D-galactoside (Sigma) was added and the reaction was stopped by the addition of 0.5 ml of 1 M Na_2_CO_3_. The optical density was measured at 420 nm. The protein concentration was measured using Bio-Rad assay and specific activities were expressed in units of β-galactosidase per milligram of protein (Miller units). Results are means of three independent experiments. Statistical values are calculated using the Student’s *t*-test and Mann–Whitney test.

### Protein Precipitation

Bc 407, Δ*nprA*, and Δ*inhA1* strains were cultured in NYB medium and taken at the indicated times after the entry into stationary growth phase. Immediately after harvesting, cultures were centrifuged at 8000 rpm for 10 min at 4°C and supernatants were filtered through a low binding protein membrane (PVDF, 0.22 μm; Millipore Company). Proteins were then precipitated twice using the deoxycholate-tetrachloroacetic acid (DOC-TCA) method (Gohar et al., 2002). Proteins were loaded onto a 12% SDS-PAGE and gels were stained using Coomassie blue staining G-250.

### Two-Dimensional Gel Electrophoresis

Two-DGE was performed as described previously (Gilois et al., 2007). Briefly, the first migration was performed on immobilized pH gradient (IPG) strips (17 cm in length, pH gradient 4 to 7, Bio-Rad) loaded with 300 mg proteins. The second migration occurred on 10 to 15% gradient SDS-PAGE gels. The gels were then stained with Coomassie blue for protein identification.

In some cases, major bands were excised from the gel and subjected to trypsin (Promega) digestion for peptide mass fingerprinting by mass spectrometry as previously described (Gohar et al., 2002).

### Purification of NprA and InhA1 Proteins

The plasmid pGEX6P1–GST–NprA was constructed as follows. The *nprA* gene was amplified from the Bc 407 chromosome by PCR using the primer pairs NprA–GST-1 (5′-CGGGATCCGATTCTAAAAATGTACTCTCTACGAAGA-3′) and NprA–GST-2 (5′-TCCCCCGGGTGAAGCGAAGTGGATTGTAACA-3′). The DNA fragment was inserted between the *Bam*H1 and *Sma*1 sites of plasmid pGEX6P1 (GE Healthcare), and the resulting plasmid was introduced into *Escherichia coli* M15 [pREP4] (Qiagen).

*Escherichia coli* M15 [pREP4] strain harboring the plasmid pGEX6P1–GST–NprA was grown at 37°C until OD_600_ 0.8 was reached and protein expression was induced by addition of 1 mM IPTG. Growth was continued for 4 h after IPTG induction. Bacteria were then collected by centrifugation at 7700 *g* for 10 min. Pellets were lysed using 1% Triton X-100 and sonication. The lysate was then centrifuged at 8000 *g* for 30 min at 4°C. Pellets were treated with 6 M urea for 1 h, then centrifuged at 25000 *g* for 40 min at 4°C. The supernatant was collected and dialyzed against 50 mM Tris, Urea 1 M, pH 8 twice and finally dialyzed against 50 mM Tris, pH 8, for 4 h at 4°C.

The tagged protein was then purified using the Bulk GST Purification module (GE Healthcare) according to the manufacturer’s instructions. The GST tag was removed by addition of PreScission protease (Sigma). Protein concentration was quantified by the Bradford method.

The complete *inhA1* gene was cloned by PCR amplification from the Bc 407 chromosome, using the primers Sin14′ and Sin15. Sin14′ (5′-CATG CCATGG CCTCTATGGAAATTATAAATTG-3′) creates an *Nco*I site, and is complementary to sequences upstream from *inhA1*. Sin15 (5′-CG GGATCCCACTATTTTTATCCAGTTC-3′) creates a *BamH*I site, and is complementary to sequences downstream from *inhA1*. The PCR product (a 2.46 kb DNA fragment) was purified, digested by *Nco*I and *BamH*I, and inserted into the vector pIVEX2.4 (Roche) and the resulting plasmid was introduced into *E. coli BL21.*

*Escherichia coli BL21* strain harboring the plasmid pIVEX–N-ter His-InhA1 was grown at 37°C until OD_600_ 0.8 was reached and protein expression was induced by addition of 1 mM IPTG and 1 mM CaCl_2_. Growth was continued for 4 h after IPTG induction. Bacteria were then collected by centrifugation at 7700 *g* for 10 min. Pellets were lysed using sonication. The lysate was then centrifuged at 8000 *g* for 30 min at 4°C. The supernatant was collected and dialyzed against 50 mM Tris, pH 8, for 4 h at 4°C. The tagged protein was then purified and eluted using the Ni-NTA resin method (Qiagen) according to the manufacturer’s instructions. Protein concentration was quantified by the Bradford method.

### Cleavage Test

Thirty ng of purified InhA1 were mixed with 2 μg of purified NprA (60 kDa) for 0 to 4 h at 30°C. Reaction was stopped by adding sample buffer and heating at 95°C for 5 min. Proteins were loaded onto a 12% SDS-PAGE and the gel was stained with Coomassie blue straining.

### N-Terminal Sequencing

The proteins of interest were extracted from 1D or 2D gels and concentrated on a PVDF-disk with the ProSorb system. The chemical process used to determine the amino acid sequence is based on Edman degradation. The identification of the NprA sequence was carried out using the automatic microsequencer (Procise Protein Sequencing System Model 494) connected to an amino acid analyzer-PTH Model 140 from Perkin Elmer Applied Biosystems.

### Cell Culture and Macrophage Infection

The murine macrophage-like cells, J774, were maintained in RPMI-1640 medium (Invitrogen) supplemented with 10% fetal bovine serum (FBS, Invitrogen) and 50 U ml^-1^/50 mg ml^-1^ penicillin/streptomycin (VWR). The cells were cultured at 37°C in 5% CO_2_ atmosphere and saturating humidity. The day prior to use, cells were detached by gentle scraping, counted with a hematocytometer and seeded into multiwell plates. For infection experiments, the day after seeding, cells were cultured in fresh serum-free medium before use in the various assays (Tran et al., 2010).

Spore release infection experiments were performed as previously described (Ramarao and Lereclus, 2005). Briefly, spores were added at a multiplicity of infection (moi) of 10. Phagocytosis was allowed to proceed for 30 min at 37°C in freshly added RPMI-1640 without serum. Non-attached spores were removed by extensive washing with PBS. The cells were covered with RPMI and incubated further at 37°C under a 5% CO_2_ atmosphere. After 0–6 h, spores or bacteria and cells were visualized by light microscopy. The amount of cells containing at least one intracellular bacteria were counted under the microscope. Results are mean values from at least three independent experiments done in duplicate. Error bars correspond to standard deviations. *P*-values were calculated using the Student’s *t*-test.

## Author Contributions

NR conceived the study, performed the experiments, analyzed the data, and wrote the manuscript. AH, S-LT, EG, CD, SP, LC, and TJ performed the experiments. DL analyzed data.

## Conflict of Interest Statement

The authors declare that the research was conducted in the absence of any commercial or financial relationships that could be construed as a potential conflict of interest.
